# Book Review

**Published:** 1911-12

**Authors:** 


**The Essentials of Histology.** By E. A. SCHÄFER, M.D., Sc.D. Eighth Edition. Pp. xi, 571. London: Longmans, Green and Co. 1910.—This textbook has by sheer merit forced its way to the front, and is used in almost every medical school in English-speaking lands. The new edition has been more fully illustrated, and brought well up to date. The most interesting of the additions refer to the structure of the pituitary gland, and to various types of unipolar nerve-cells, here following the important researches of Ramon y Cajal.

**Acute Intestinal Toxæmia in Infants.** By RALPH VINCENT, M.D. London: Baillière, Tindall & Cox. 1911.—The whole burden of this address, delivered before the Glasgow Obstetrical and Gynæcological Society, is a plea for the use of unboiled milk in the feeding of infants. In the last chapter Dr. Vincent says, "The dirtier the milk, the more important is it that it should not be cooked." A series of experiments with kittens fed on boiled milk are devised to prove the author's contention that heated milk, owing to the destruction of the lactic acid forming organisms, offers a ready pabulum for the growth of pathogenic germs. We consider the views put forward are opposed to accepted teaching and contrary to experience, and if put into practice in their entirety would tend to an enormous increase in infant mortality.

**A Study of the After-Results of Abdominal Operations on the Pelvic Organs.** By ARTHUR E. GILES, M.D., B.Sc., F.R.C.S. Pp. viii., 251. London: Baillière, Tindall & Cox. 1910.—The conclusions which the author draws in his book are based on 1,000 consecutive cases, and the work is a reprint of a series of articles which appeared in the *Journal of Obstetrics and Gynæcology of the British Empire.* The first portion—about two-fifths—of the book deals with the answers to questions which may arise when we consider the after-results of operations, the rest of the volume consists of tables composed of a brief account of the individual cases upon which the writer's conclusions are founded. Many interesting points are discussed in the first part of the book, *e.g.* after removal of the appendages of one side, what is the value of the remaining ovary from the point of view of subsequent pregnancy? How is the general health affected after hysterectomy for fibroids? and so forth. The mortality of many operations is so low that it is rather to the after-results that we must look in order to justify us in choosing or refusing surgical interference in many cases which might be classed as expedient rather than necessitous.

**Sprains and Allied Injuries of Joints.** By R. H. ANGLIN WHITELOCKE, M.D., M.C., F.R.C.S. Second Edition. Pp. xix, 280. London: Oxford Medical Publications. 1910.—This is a useful book, full of practical information, but its title is hardly comprehensive enough, for injuries of other structures than joints are considered in it. For instance, injuries of muscles and tendons occupy the space of two chapters. We rather object to the names "fracture-sprain" and "dislocation-sprain." In all fractures and dislocations there may or may not be extensive injury to the soft parts, but if there is a definite fracture or dislocation let it be called so. The cases described under these names are those in which the signs of fracture or dislocation might not be very evident until a skiagram is taken, but we do not think that therefore they differ in their nature from more evident fractures and dislocations. Perhaps the author has called them fracture-sprains to bring them within the scope of his book. We would suggest that it might be better simply to point out how they are to be distinguished from simple sprains. Of course, internal derangements of the knee-joint form an important subject for consideration. The illustrations which show the author's method of reducing displacement of the semilunar cartilages of the knee are most helpful in showing the method employed, and the book is, as a whole, really well illustrated, with quite useful photographs and skiagrams. The illustrations of myositis ossificans are particularly useful. No doubt practising in Oxford, where there is much athletic exercise amongst the undergraduates, he has experience of many forms of injury and thus has been able to write a really helpful book. Schlater's disease is fully considered, and some illustrative cases, with skiagrams, are given. It might be well to include an account of "snapping hip" in the next edition. It is a very puzzling condition, and is very apt to be considered traumatic in its origin by the patient, and of the nature of a partial dislocation even by the surgeon. We can commend this book to all those who have many of the slighter accidents to treat.

**Handbook of the Surgery of the Kidneys.** By W. BRUCE CLARKE. Pp. x, 199. London: Oxford Medical Publications. 1911.—This book is presented in a form suggesting a series of clinical lectures, recording chiefly the author's personal experience and views on the subject. It is intended, as is stated, as a handbook for the busy practitioner, and as such deals somewhat superficially with the subject, but from this point of view contains numerous useful hints on the diagnosis, treatment and prognosis of surgical diseases of the kidney.

**New and Non-official Remedies, 1911.** Chicago: American Medical Association. 1911.—This is the 1911 edition of the annual *New and Nonofficial Remedies* issued by the Council on Pharmacy and Chemistry of the American Medical Association, and contains descriptions of all articles approved by the Council up to Dec. 31st, 1910. There are also descriptions of a number of unofficial non-proprietary articles which the Council deemed of value. In the arrangement and the scope of individual descriptions the present edition does not differ widely from the 1910 edition, but it contains about twenty-five additional pages, these being required to describe the articles accepted by the Council during 1910. Besides indicating to physicians the proprietary articles which the Council's examination has found to be honestly marketed, and containing accurate descriptions of these articles, all similar articles are arranged under group headings; thus the physician at a glance can learn that atoxyl and soamin are practically identical articles, and that arsacetin is a closely related body. Again, the several proprietary solutions of the blood-pressure-raising principle of the suprarenal gland are listed under a general title "epinephrin," and the manner in which the solutions differ from each other can be learned at a glance. In the same way, the medicinal foods are brought together and their relative value compared.

**Golden Rules of Refraction.** By Ernest E. Maddox, M.D., F.R.C.S.E. Third Edition. Pp. 96. Bristol: John Wright & Sons Ltd. (n.d.)—This book is entirely practical in its scope. It is not a "cram" book, but to those who have acquired some knowledge of refraction from the larger text-books and practice it will be found to be full of an extraordinary number of valuable hints, particularly on little practical points often not to be found in the larger works. It has reached a third edition, and so has evidently filled a want.

**Hygiene and Public Health.** By Louis C. Parkes, M.D., D.P.H., and Henry R. Kenwood, M.B., F.R.S. Edin., D.P.H. Fourth Edition. Pp. xi, 691. London: H. K. Lewis. 1911. —This well-known handbook, which had run through five editions before it came under the joint authorship of Drs. Parkes and Kenwood, maintains its position as a concise, readable and reliable guide to students of Public Health. In the fourth edition the pages number 691 compared with the 620 of the third edition, and the additional pages have been well utilised in incorporating recent discoveries of importance, not forgetting even "606." The subject of school hygiene is not neglected, and the chapters on causation of the communicable and epizootic diseases have been revised. Possibly the introductory section on " The Contagia " might be recast so as to give the protozoa their due prominence in disease causation ; and it seems hardly necessary to-day to be so apologetic with regard to the " germ theory." Taken altogether, this is one of the most useful of students' handbooks.

**Transactions of the Association of American Physicians.** Vol. XXV. Philadelphia. 1910.—The usual high scientific and literary standard is amply maintained in the latest volume of these *Transactions*. Want of space prevents our referring to the various contributions in detail. Rotch and Smith's paper, however, on the determination of the developmental progress of adolescents by radiographic examination of the epiphyses of the wrists strikes us as full of promise in many directions. It involuntarily recalls the remarkable address of Sir James Paget on " Errors in the Chronometry of Life." We cannot help noticing that while our common language seems to tend to an ever-increasing separation in style and idiom in the mouths of the vulgar, American and English physicians remain still content with a language which is mutually intelligible.

**How to Keep Fit.** By A. T. Schofield, M.D., M.R.C.S. Pp. 79. London : William Rider & Son Ltd. 1910.—This little book belongs to a series called " Rider's Mind and Body Handbooks." It is addressed to the public, is written in a popular style, and contains general advice and maxims on hygiene, including paragraphs on smoking, tea-drinking, dress, fresh air, rest, feeding, and so forth. It is sensibly written, and may very well be placed in the hands of anyone who wants a little advice on such questions. It is full of platitudes, but these are often put in a forcible and convincing manner. There is a slight tendency to be dogmatic, but probably this is useful when talking to the ignorant. Sometimes a statement occurs one can hardly agree with, such as " no healthy child will eat or sleep too much." But most of the book is common-sense and correct. The author is at his best when denouncing the habit of continuous contemplation of one's ailments, and he strongly advises his readers to avoid introspection and self-analysis.

**A Manual of Nursing.** By Margaret Frances Donahoe. Pp. x, 489. London : D. Appleton & Company. 1910.—This book is more readable than many of the manuals on nursing. It has the advantage of not going too deeply into anatomical and physiological details. It is evenly balanced, and covers all the necessary ground. Massage and midwifery are not included, and there is no special chapter on private nursing.

The chapter on bandaging is too condensed, and might well have been omitted. The description of the various diseases are short and intelligible, without giving too much detail. It may be recommended to nurses to give them a very fair idea of what is expected of them.

**Transactions of the College of Physicians of Philadelphia.** Vol. XXXII. Philadelphia: Printed for the College. 1910.—The high standard which we have learnt to expect of these *Transactions* is fully maintained. The volume is full of good and varied reading. It is difficult to estimate the full value of researches in experimental medicine when the work is first accomplished, but we cannot but regard Pemberton and Sweet's paper on the " Induction of Pancreatic Activity by the Removal of the Adrenals " as a contribution which should command the attention of physicians no less than physiologists and pathologists. Among their conclusions one stands out prominently, namely the suggestion of a control over the pancreas by the adrenals in the absence of which the gland secretes more actively. " A Study of the Cammidge Reaction," by Swan and Gilbride, contributes to the increasing weight of evidence against the reliability of this reaction. Every surgeon will be interested in the symposium on Post-operative Psychoses and Insanities, opened by Dr. Weir Mitchell. There is an excellent series of papers on Epidemic Poliomyelitis, and some useful observations on the pathological changes in Tabes Dorsalis by Rhein.

**Medical Revolution.** By SYDNEY W. MACILWAINE, M.R.C.S., L.R.C.P. (retired). London: P. S. King & Son. 1911.—The book professes to be a plea for the national preservation of health based upon the natural interpretation of disease, and it is dedicated to " the intelligent, and puzzled, layman, to medical officers of health, and general practitioners, and to medical students, to whom belongs the future of our profession." The author, this superior person, appears to take an elevated position from which he criticises adversely everything which is. The whole field is beset with errors in nomenclature, pathology, diagnosis and illusory diagnosis, false relations of theory and practice, errors in the hospital, errors in medical organisation and education, errors in certification, and errors even in quackery and empiricism. The book itself abounds in inaccuracies, and is a good illustration of the author's contention that the ideal is difficult of attainment. We may well summarise it in the words " much cry and little wool."

